# Central Adiposity Indicators Maintain a Stronger Association With the Risk of Hypertension: A Prospective Cohort Study in Southwest China

**DOI:** 10.3389/ijph.2022.1605305

**Published:** 2022-10-05

**Authors:** Tao Liu, Yawen Wang, Ningxin Gao, Jie Zhou, Yiying Wang, Chaowei Fu, Kelin Xu

**Affiliations:** ^1^ Guizhou Centre for Disease Control and Prevention, Guiyang, China; ^2^ Department of Biostatistics, School of Public Health, Fudan University, Shanghai, China; ^3^ Key Laboratory of Public Health and Safety of the Ministry of Education, Fudan University, Shanghai, China; ^4^ Key Lab of Health Technology Assessment, Ministry of Health, Fudan University, Shanghai, China; ^5^ Department of Epidemiology, School of Public Health, Fudan University, Shanghai, China

**Keywords:** risk factors, cohort study, obesity, hypertension, anthropometric indices

## Abstract

**Objectives:** Studies have linked obesity to an increased risk of hypertension, but the optimal adiposity indicators to predict hypertension remains controversial. We comprehensively explored the correlation between body mass index, waist circumference (WC), waist-to-height ratio (WHtR), long-term weight and WC change, and hypertension in an adult population in Southwest China.

**Methods:** We studied 9,280 participants from 48 townships of 12 districts with a follow-up of 10 years in the Guizhou Population Health Cohort Study. We used Pearson’s correlation coefficients combined with Dunn and Clark’s z test and Zou’s confidence interval test, receiver operating characteristic (ROC) analyses, and multivariate Cox proportional hazards regressions adjusting for demographic characteristics, lifestyle habits, disease history, and lipid information of participants.

**Results:** Baseline central adiposity indicators (WC and WHtR) had closer associations with hypertension than BMI, and long-term WC change was more predictive of hypertension compared with weight change in the studied population.

**Conclusion:** Central adiposity indicators maintain a stronger association with the risk of hypertension, hinting at the importance of WC management in the precaution of hypertension.

## Introduction

Hypertension is a major cause of premature death worldwide [1]. The high prevalence and burden of hypertension, which affects the health and living qualities of 1.13 billion people worldwide [2], is a serious concern [3–5]. Two-thirds of adults aged 30–79 years with hypertension worldwide live in low- and middle-income countries, mainly due to increased risk factors in those populations in recent decades [1–3, 6]. Reducing modifiable risk factors, including being overweight or obese, is among the best ways to prevent hypertension and related diseases and organ damage, and avoiding dietary and behavioral risk factors is doubly essential for those with unmodifiable or hereditary risk factors [7].

Evidence has demonstrated the association between obesity and an increased risk of hypertension [8–12]. Data from NHANES indicated that obesity is an important predictor of elevated blood pressure [8]. Although the association between hypertension and obesity has been confirmed, the best anthropometric index of obesity that predicts or associates strongly with hypertension remains controversial. Some studies have observed that waist circumference (WC) maintained a stronger association with hypertension than body mass index (BMI) [13–15], while other studies found that BMI best predicted hypertension [16, 17]. Additionally, the Korean Genome and Epidemiology Study indicated that the central obesity indices like WC and waist-height ratio (WHtR) were better than BMI for the prediction of hypertension in middle-aged Korean people [18]. And there is evidence indicating WHtR had the best performance for discriminating hypertension [19–21]. Besides, a meta-analysis done exclusively on the Caucasian population concluded that compared to WHtR, WC was more associated with hypertension [13]. Moreover, few studies assessed the predictive abilities of long-term weight or WC change for hypertension. It is necessary to compare the effects of long-term weight or WC change on hypertension as it accumulated over time.

Therefore, the main objective of this study was to compare the association of commonly used baseline adiposity indicators (BMI, WC, and WHtR) and long-term adiposity changes (weight and WC change from baseline to follow-up) with hypertension in an adult population in Southwest China, and to comprehensively assess the predictive power of adiposity indicators for hypertension.

## Methods

### Study Design and Population

The Guizhou Population Health Cohort Study (GPHCS) is a community-based prospective cohort study in Guizhou Province, China. A total of 9,280 adult residents in 48 townships of 12 districts in Guizhou Province were recruited from 2010 to 2012 using the multi-stage proportional stratified cluster sampling method. Inclusion criteria were [1]: 18 years of age or older [2]; living in the study area with no plans to move out [3]; completing the survey questionnaire and taking blood samples; and [4] signing a written informed consent. This study was approved by the Institutional Review Board of the Guizhou Provincial Center for Disease Control and Prevention (No. S2017-02). All subjects provided written informed consent at the time of enrollment into the cohort.

All participants were followed up through repeat surveys between 2016 and 2020 to collect information on major chronic conditions and vital status. All deaths were confirmed through the Death Registration Information System and the Basic Public Health Services System. In our analysis, we excluded 1) subjects who had a history of hypertension at baseline (*n* = 2,132), 2) subjects who were lost to follow-up (*n* = 1,117), 3) subjects with missing hypertension data at follow-up (*n* = 406), and 4) subjects with missing height or weight information at baseline (*n* = 12). Finally, a total of 5,613 participants were included in our analysis.

### Anthropometric Indicators of Obesity

In this study, we investigate anthropometric indices of obesity including body mass index (BMI), waist circumference (WC), waist-height ratio (WHtR), weight change, and WC change. Participants’ standing height, body weight, and WC were measured by trained investigators, both at baseline and follow-up. Standing height was measured using a portable stadiometer to the nearest 0.1 cm and body weight was measured using digital weight scales to the nearest 0.1 kg. WC was measured at the midpoint between the lowest rib margin and the iliac crest to the nearest 0.1 cm. BMI was calculated as body weight (kg) divided by the square of height (m^2^). BMI was evaluated using either per standard deviation (SD) increase or divided into four categories (low normal weight <22.0; high normal weight 22.0–23.9; overweight 24.0–27.9; and obese ≥28.0 kg/m^2^) according to the Chinese BMI classification criteria [22]. WC was evaluated using either per SD increase or divided into two categories (normal WC < 85 cm for women and <90 cm for men; central obesity ≥85 cm for women and ≥90 cm for men) [23]. WHtR was calculated as WC (cm) divided by height (cm) and evaluated by either per SD increase or divided into two categories (low WHtR <0.5 and high WHtR ≥0.5) [24]. The amount of weight change in participants was calculated by subtracting the weight recorded at baseline from the weight recorded at the follow-up minus and evaluated by either per SD increase or divided into four categories according to its distribution (loss of >2 kg, loss of ≤2 to gain of <2 kg, gain of ≥2 to gain of <6 kg, and gain of ≥6 kg). The amount of WC change in participants was calculated by subtracting the WC recorded at baseline from the WC recorded at the follow-up minus and evaluated by either per SD increase or divided into four categories according to its distribution (loss of >3 cm, loss of ≤3 to gain of <3 cm, gain of ≥3 to gain of <9 cm, and gain of ≥9 cm).

### Hypertension

Participants were diagnosed with hypertension if they met either of the following two criteria [1]: self-reported diagnosis of hypertension or anti-hypertensive treatment by physicians; or [2] systolic blood pressure (SBP) ≥140 mmHg and/or diastolic blood pressure (DBP) ≥90 mmHg [25]. The blood pressure was measured with the same type of electronic sphygmomanometer and was accurate at 0.1 mmHg. If the difference between the three measurements did not exceed 10 mmHg, the average of the three measurements was taken as the final reading; if the difference between the three measurements was large, the average of the two similar measurements was taken as the final reading; if only one measurement was taken, the final reading was taken directly.

### Covariates

All participants were interviewed face-to-face and completed a structured questionnaire that provided information relating to participants’ demographic characteristics (sex, age, area, ethnicity, marital status, and occupation), lifestyle habits (smoking status, alcohol use, and exercise), and history of diabetes. Participants were diagnosed with Type 2 diabetes mellitus (T2DM) if they met any of the following criteria [1]: self-reported diagnosis of diabetes or anti-diabetic treatment by physicians [2]; fasting plasma glucose (FPG) of ≥7.0 mmol/L [3]; oral glucose tolerance test (OGTT) result of ≥11.1 mmol/L; or [4] hemoglobin A1c (HbA1c) of ≥6.5% [26]. Venous blood samples were collected after an overnight fast of at least 8 h to measure total cholesterol (TC), triglycerides (TG), high-density lipoprotein cholesterol (HDL-C), and low-density lipoprotein cholesterol (LDL-C).

### Statistical Analysis

Continuous variables were represented by mean (SD) and categorical variables by percentages. The Student’s t-test for continuous variables and the Chi-square test for categorical variables were done to compare the differences between new hypertension cases and non-hypertension subjects. The follow-up person-years (PYs) were calculated from the date of enrollment in the cohort to the date of hypertension diagnosis, death, or follow-up, whichever occurred first.

Associations between anthropometric measures and hypertension were first measured by Pearson’s correlation coefficients and the corresponding 95% confidence intervals. Pearson’s correlation coefficients were classified into four categories: very strong (0.90–1.00), strong (0.70–0.89), moderate (0.40–0.69), weak (0.10–0.39), and negligible (0–0.10) [27], to present the strength of the association. To control for the possibility of an observed difference between two correlations occurring simply by chance, a test of significance is necessary. Hittner, May, and Silver’s modification of Dunn and Clark’s z test and Zou’s confidence interval test were utilized to compare two overlapping correlations in dependent groups [28].

Receiver Operating Characteristic (ROC) analyses were then used to compare differences in the prediction of hypertension across anthropometric measures [29–31]. The area under the ROC curve (AUC) and the corresponding 95% confidence intervals for half of the follow-up person-years were determined by the time-dependent ROC curve. In addition, time-AUC curves of various anthropometric measures were utilized to compare the AUC at each time point.

Finally, multivariate Cox proportional hazards regression model was performed to estimate the association between hypertension and baseline general adiposity indicator (BMI), central adiposity indicators (WC and WHtR), and long-term adiposity changes (weight and WC change from baseline to follow-up). We fitted three separate models of the association between each obesity indicator and hypertension and tested the anthropometric measures individually in separate Cox models [1]: Model 1: adjusted for age (as continuous) and sex [2]; Model 2: Model 1 plus area (urban, rural), ethnicity (ethnic minorities, the Han nationality), marriage (married, unmarried, other), occupation (farmer, unemployed, and retired, other), smoking status (current smoker, non-current smoker), alcohol use (yes or no), exercise (yes or no), and history of diabetes (yes or no) [3]; Model 3: Model 2 plus SBP, total cholesterol, triglycerides, HDL-C value, LDL-C value, and baseline BMI value (in the analyses of weight change and WC change). The adjusted hazard ratios (HRs) were presented with 95% confidence intervals (CIs). The proportional hazards assumption in Cox regression models was tested using Schoenfeld residuals, and no evidence of non-proportionality was observed. In addition, we fitted multivariate Cox models with restricted cubic splines with 4 knots at the 5th, 35th, 65th, and 95th percentiles to test for linearity in the association between anthropometric indices of obesity and incident hypertension and found no evidence of nonlinearity (Supplementary Table S1). We also calculated the C-index to evaluate the model performance of these Cox models adjusted for the covariates described above.

The robustness of our findings was assessed in multiple sensitivity analyses. First, we re-estimated the association between obesity indicators and the risk of hypertension after excluding the participants who followed up in less than 1 year. Second, we calculated E-values to explore the possibility of unmeasured confounding between obesity indicators and hypertension risk [32, 33]. The E-value quantifies the required magnitude of unmeasurable confounders that may negate the currently obtained correlation between obesity indicators and the risk of hypertension.

All analyses were two-tailed and *p* < 0.05 was considered to indicate statistical significance. All analyses were conducted in R software (version 4.1.0).

## Results

### Baseline Characteristics

The basic characteristics of the study population are presented in Supplementary Table S2. A total of 5,613 participants were included in the study, and 1,214 (21.6%) were diagnosed with hypertension at follow-up. The mean age of the study population was 42.02 ± 14.17 years and most were women. Participants were more likely to be married, to be farmers, to be exercise population, and had a lower proportion of ethnic minority. Only 6.3% of subjects had a history of diabetes, and a small number of subjects were current drinkers and smokers. Compared with participants who remained free of hypertension, new hypertension cases were older and had a significantly higher mean BMI, WC, WHtR, SBP, and TG.

In addition, we also found that of the 9,280 participants, 1,117 (12.04%) were lost to follow-up. Compared to the complete follow-up group, subjects lost to follow-up were older at baseline and had statistically significant differences in mean BMI, SBP, TC, and LDL. The detailed results of the comparison were shown in Supplementary Table S3.

### Correlations Coefficients

**FIGURE 1:**
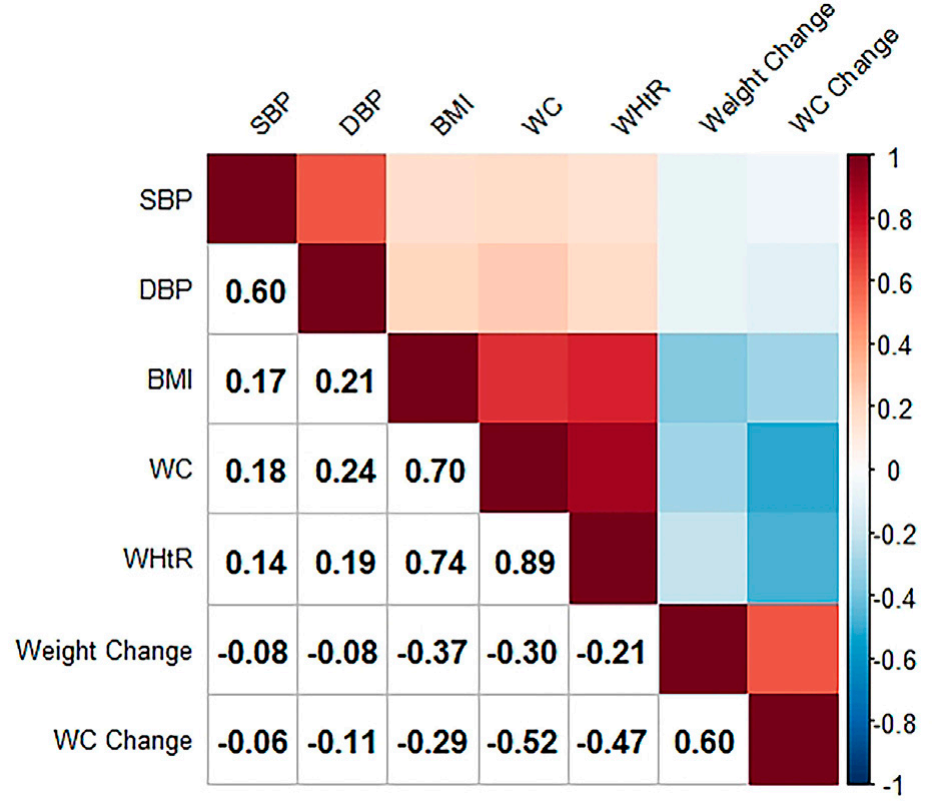
Intercorrelations between anthropometric indices and blood pressure (The Guizhou population health cohort study, China, 2010–2012). SBP, systolic blood pressure; DBP, diastolic blood pressure; BMI, body mass index; WC, waist circumference; WHtR, waist-to-height ratio.

The Pearson’s correlation coefficients between the various anthropometric measures with blood pressure are displayed in Figure 1 and Supplementary Table S4. Baseline BMI, WC, and WHtR were positively and weakly correlated with blood pressure (*p* < 0.001), among which WC correlated with blood pressure most closely (both SBP and DBP were considered). Weight change and WC change from baseline to follow-up were negatively correlated with blood pressure (*p* < 0.001). Compared with WC change, Weight change had a higher but negligible correlation with SBP and a lower correlation with DBP. We further compared these associations and found that the correlation between DBP and WC was significantly higher than the other two associations, and WC change had a significantly larger negative effect on DBP compared with weight change. Moreover, the correlation with blood pressure (both SBP and DBP) was significantly higher for WC than for long-term adiposity changes (weight change and WC change from baseline to follow-up). The detailed results of the association comparison were listed in Table 1.

**TABLE 1:** Test results of comparisons of two overlapping correlations in dependent groups (The Guizhou population health cohort study, China, 2010–2012).

	Hittner2003^a^	Zou2007^b^
	*p*-value	95% CI^d^
r (SBP^d^, WC^d^) vs. r (SBP, BMI^d^)	0.93	(−0.02, 0.02)
r (SBP, WC) vs. r (SBP, WHtR^d^)	**0.00** **^c^**	**(0.02, 0.04)**
r (SBP, WC Change) vs. r (SBP, Weight Change)	0.08	(−0.05, 0.00)
r (SBP, WC) vs. r (SBP, Weight Change)	**0.00**	**(0.21, 0.31)**
r (DBP^d^, WC) vs. r (DBP, BMI)	**0.00**	**(0.01, 0.05)**
r (DBP, WC) vs. r (DBP, WHtR)	**0.00**	**(0.04, 0.06)**
r (DBP, WC Change) vs. r (DBP, Weight Change)	**0.03**	**(**−**0.06, -0.00)**
r (DBP, WC) vs. r (DBP, WC Change)	**0.00**	**(0.31, 0.41)**

a Hittner, May, and Silver’s (2003) modification of Dunn and Clark’s z (1969) using a back-transformed average Fisher’s (1921) Z procedure, which is a significance test.

b Zou’s (2007) confidence interval is a test based on the computation of confidence intervals.

c Results that reject the null hypothesis are marked in **bold**.

d SBP, systolic blood pressure; DBP, diastolic blood pressure; BMI, body mass index; WC, waist circumference; WHtR, waist-to-height ratio; 95% CI, 95% confidence interval.

### Receiver Operating Characteristic Analyses

**FIGURE 2:**
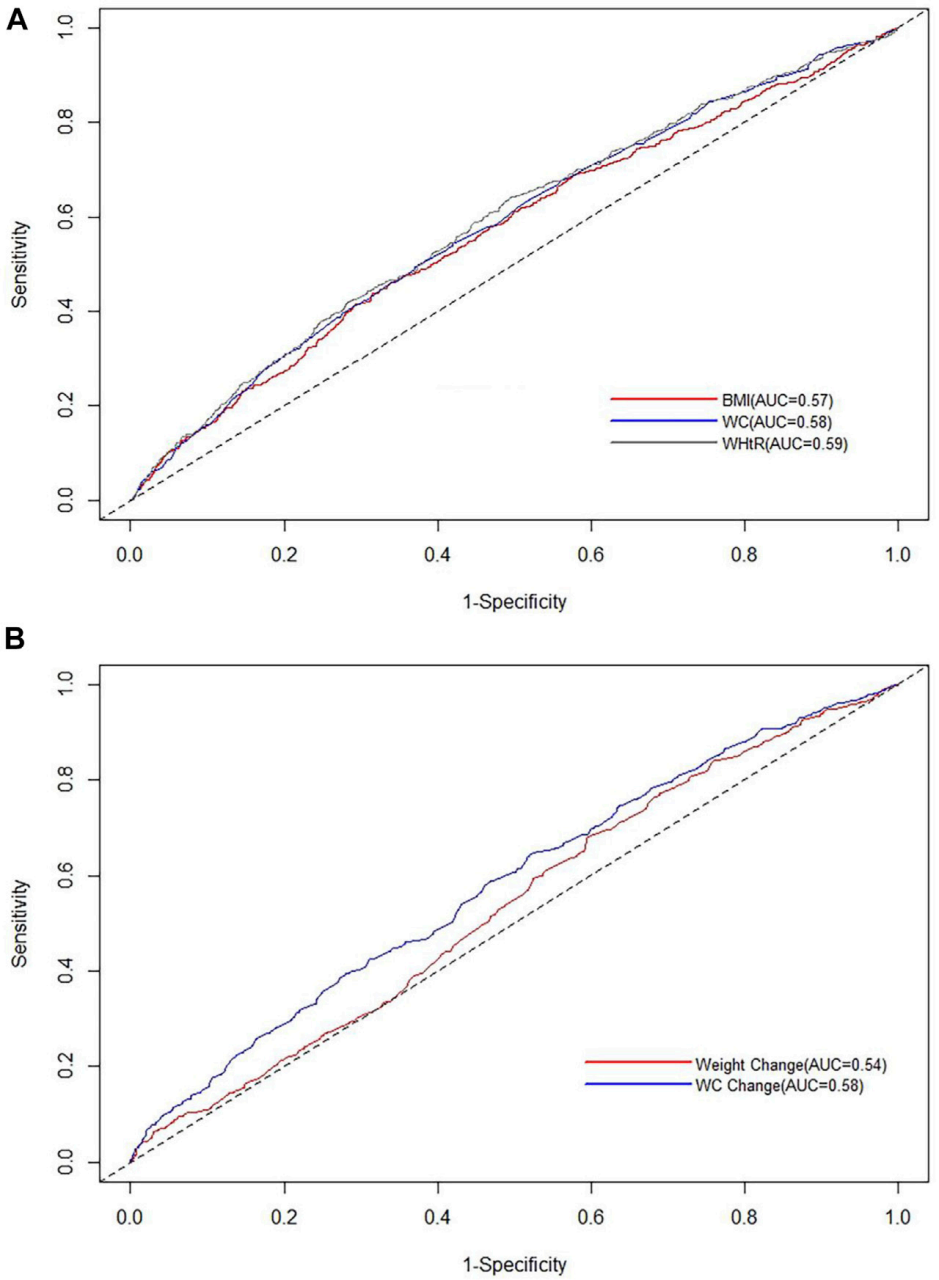
Time-dependent receiver operating characteristic curves for half of the follow-up person-years of various anthropometric indices for hypertension (The Guizhou population health cohort study, China, 2010–2012). **(A)** Time-dependent receiver operating characteristic curves for half of the follow-up person-years of baseline body mass index, waist circumference, and waist-to-height ratio for hypertension; **(B)** time-dependent receiver operating characteristic curves for half of the follow-up person-years of weight change and waist circumference change from baseline to follow-up for hypertension. BMI, body mass index; WC, waist circumference; WHtR, waist-to-height ratio.

The time-dependent ROC curves for half of the follow-up person-years and AUCs of various anthropometric indices for hypertension were shown in Figure 2 and Supplementary Table S5. We found WHtR had the largest AUC for hypertension, but the differences in the AUC for the baseline adiposity indicators were small with overlapping 95% confidence intervals. However, we still found the AUC for WC change from baseline to follow-up was significantly higher than weight change. In addition, we further compared the AUC of WHtR to WC change at half of the follow-up person-years and found a greater AUC for WHtR compared to WC change, but the results were not significant due to overlapping 95% confidence intervals. Detailed information was shown in Supplementary Table S5 and Supplementary Figure S1.

**FIGURE 3:**
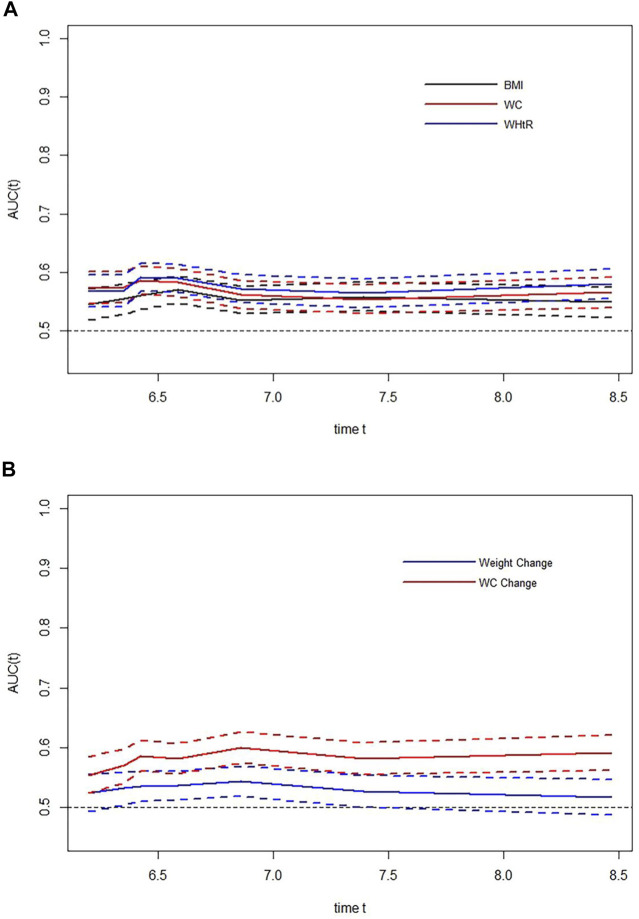
Time-dependent areas under curves of various anthropometric indices for hypertension (The Guizhou population health cohort study, China, 2010–2012). **(A)** Time-dependent areas under curves of baseline body mass index, waist circumference, and waist-to-height ratio for hypertension; **(B)** time-dependent areas under curves of weight change and waist circumference change from baseline to follow-up for hypertension. BMI, body mass index; WC, waist circumference; WHtR, waist-to-height ratio; AUC, areas under the receiver operating characteristic curves.

The time-AUC curves between each anthropometric measure and hypertension were displayed in Figure 3. Time-AUC curves showed that WHtR had the slightly largest AUC among the three anthropometric indices, while the AUC of WC change from baseline to follow-up for hypertension was significantly higher than the AUC of weight change when the follow-up time exceeds 6.5 years. And the effect of WC change on hypertension was stronger than that of weight change as it accumulated over time. However, the differences in the AUC for baseline WHtR, BMI, and WC were not statistically significant with overlapping 95% confidence intervals. Additionally, we found WC change from baseline to follow-up had a greater AUC than WHtR after approximately 6.7 years of follow-up, but the results were not significant due to overlapping 95% confidence intervals. Time-AUC curves of WHtR and WC change were placed in Supplementary Figure S2.

### Hazard Ratios of Hypertension by Anthropometric Measures

As shown in Table 2, baseline BMI, WC, and WHtR were associated with incident hypertension in the study population. Compared with the normal weight subjects (BMI: 22.0–23.9 kg/m^2^), obese participants (BMI: ≥28.0 kg/m^2^) had a statistically increased risk of hypertension, with fully adjusted HRs of 1.50 (95% CI: 1.16–1.93). Central obesity (WC ≥ 85/90 cm) increased the risk of hypertension in participants by 33% (HR = 1.33, 95% CI: 1.12–1.57), after adjustment for potential covariates. Participants with high WHtR (≥0.5) had a 25% increased risk of hypertension compared to subjects with low WHtR (<0.5). For per SD increase in BMI, WC, and WHtR, the risk of hypertension increased by 10%, 15%, and 14%, respectively, in Model 3. We found that WC had the highest HRs for hypertension among all the anthropometric measures. However, HRs were not considered significantly different from each other due to the overlapping 95% confidence intervals.

**TABLE 2:** Hazard ratios (95% confidence intervals) of hypertension associated with baseline body mass index, waist circumference, and waist-to-height ratio (The Guizhou population health cohort study, China, 2010–2012).

	Cases, n	HR (95% CI)^a^		
		Model 1^b^	Model 2^c^	Model 3^d^
Body mass index, kg/m^2^	5,613	1.15 (1.09, 1.21)***^e^	1.14 (1.08, 1.20)***	1.10 (1.04, 1.17)**
<22.0	2,722	0.84 (0.73, 0.97)*	0.85 (0.74, 0.98)*	0.90 (0.78, 1.04)
22.0–23.9	1,343	1.00	1.00	1.00
24.0–27.9	1,277	1.01 (0.86, 1.18)	1.00 (0.85, 1.17)	0.99 (0.84, 1.17)
≥28.0	271	1.58 (1.24, 2.03)***	1.53 (1.19, 1.96)***	1.50 (1.16, 1.93)**
Waist circumference, cm	5,266	***1.18*** (***1.11, 1.26***)***	***1.18*** (***1.11, 1.25***)***	***1.15*** (***1.08, 1.23***)***
<85/90	4,693	1.00	1.00	1.00
≥85/90	573	1.43 (1.21, 1.69)***	1.41 (1.20, 1.67)***	1.33 (1.12, 1.57)**
Waist-to-height ratio	5,266	1.17 (1.11, 1.24)***	1.17 (1.10, 1.23)***	1.14 (1.08, 1.21)***
<0.5	3,521	1.00	1.00	1.00
≥0.5	1745	1.31 (1.17, 1.48)***	1.30 (1.15, 1.46)***	1.25 (1.10, 1.42)***

***p < 0.001, **p < 0.01, *p < 0.05.

a HR, hazard ratio; 95% CI, 95% confidence interval.

b Adjusted for age (continuous variable), sex.

c Model 1 plus area, ethnicity, marriage, occupation, smoking status, alcohol use, exercise, and history of diabetes.

d Model 2 plus SBP, total cholesterol, triglycerides, HDL-C value, LDL-C value.

e The anthropometric measure (per SD increase) with the highest significant HR value was marked in **bold italics**.

We found that weight change and WC change from baseline to follow-up had a statistically increased risk of hypertension after adjustment for major covariates and baseline BMI, with fully adjusted HRs of 1.27 (95% CI: 1.19–1.35) and 1.43 (95% CI: 1.34–1.52), per SD increase (Table 3). Participants with a weight gain of ≥2 to gain of <6 kg or a weight gain of ≥6 kg had significant increased HRs for incident hypertension compared with participants who maintained baseline weight (±2 kg), which were 1.40 (95% CI: 1.16–1.70) and 1.56 (95% CI: 1.29–1.87), respectively, in Model 3. Compared with participants who maintained baseline WC (±3 cm), those with a WC gain of ≥3 to gain of <9 cm or a WC gain of ≥9 cm experienced an increased risk of hypertension with fully adjusted HRs of 1.37 (95% CI: 1.11–1.69) and 1.83 (95% CI: 1.51–2.21), respectively, in Model 3. We found that the HRs of hypertension were higher for each SD increase of long-term WC change than for each SD increase of long-term weight change. Finally, although the 95% confidence intervals were slightly overlapped, there is a trend that the effect of WC change on hypertension was larger than that of weight change.

**TABLE 3:** Hazard ratios (95% confidence intervals) of hypertension associated with weight change and waist circumference change from baseline to follow-up (The Guizhou population health cohort study, China, 2010–2012).

	Cases, n	HR (95% CI)^a^		
		Model 1^b^	Model 2^c^	Model 3^d^
Weight change, kg	4,480	1.19 (1.12, 1.26)***^e^	1.19 (1.12, 1.26)***	1.27 (1.19, 1.35)***
loss of >2	1,065	0.98 (0.81, 1.19)	0.98 (0.81, 1.19)	0.91 (0.75, 1.11)
loss of ≤2 to gain of <2	1,097	1.00	1.00	1.00
gain of ≥2 to gain of <6	1,000	1.36 (1.12, 1.64)**	1.33 (1.10, 1.61)**	1.40 (1.16, 1.70)***
gain of ≥6	1,318	1.40 (1.17, 1.67)***	1.41 (1.18, 1.68)***	1.56 (1.29, 1.87)***
WC^a^ change, cm	4,083	***1.35*** (***1.26, 1.43***)***	***1.33*** (***1.25, 1.42***)***	***1.43*** (***1.34, 1.52***)***
loss of >3	607	0.86 (0.67, 1.10)	0.85 (0.66, 1.09)	0.79 (0.61, 1.01)
loss of ≤3 to gain of <3	902	1	1	
gain of ≥3 to gain of <9	1,021	1.26 (1.03, 1.55)*	1.26 (1.02, 1.55)*	1.37 (1.11, 1.69)**
gain of ≥9	1,553	1.69 (1.41, 2.03)***	1.64 (1.36, 1.97)***	1.83 (1.51, 2.21)***

***p < 0.001, **p < 0.01, *p < 0.05.

a HR, hazard ratio; 95% CI, 95% confidence interval; WC, waist circumference.

b Adjusted for age (continuous variable), sex.

c Model 1 plus area, ethnicity, marriage, occupation, smoking status, alcohol use, exercise, and history of diabetes.

d Model 2 plus SBP, total cholesterol, triglycerides, HDL-C value, LDL-C value, and baseline BMI, value.

e The anthropometric measure (per SD increase) with the highest significant HR value was marked in **bold italics**.

As shown in Supplementary Table S6, we found that generally, C-indices of the models using the central obesity indicators were a little bit higher than those using the general obesity indicators, indicating that the model performance using the central obesity indicators was superior to that using the general obesity indicators. This finding further highlights the importance of WC management in the precaution of hypertension.

### Sensitivity Analysis

In the sensitivity analysis conducted to assess the robustness of our results, the effect estimates of baseline BMI, WC, WHtR, weight change, and WC change from baseline to the follow-up on hypertension did not change substantially after excluding subjects diagnosed with hypertension within one year of entry into the cohort (Supplementary Table S7).

We also generated E-values to assess the sensitivity to unmeasured confounding [34]. E-values were higher than the HRs of the covariates, and unmeasurable confounders could not completely negate the association effects observed in our current study. See Supplementary Tables S8–S10 for other details.

## Discussion

This study compared the association of general adiposity indicators (baseline BMI and long-term weight change) and central adiposity indicators (baseline WC, WHtR, and long-term WC change) with hypertension based on the first 10-year prospective cohort study of adults in Southwest China, and comprehensively assessed the predictive power of adiposity indicators for hypertension. Based on the results of correlation coefficients, estimated AUCs, and HR values, we found that baseline central adiposity indicators (WC and WHtR) showed a closer association with hypertension. Moreover, our results indicated that WC change from baseline to follow-up was a better predictor of hypertension than weight change in the studied population. It is important to note that these findings are still potentially biased and need to be confirmed by further studies.

Previous studies have demonstrated the association of adiposity indicators such as BMI, WC, and WHtR with hypertension and its predictive power for hypertension, but the best adiposity indicators have not reached consensus [13, 16–19, 35–39]. In view of the potential bias, the present study reported that central obesity indicators showed the best performance for predicting hypertension, that was baseline obesity indicators (WC and WHtR) have been proposed to have a closer association with hypertension than BMI [13, 18, 19, 38], and WC change presented better predictive power for hypertension than weight change. However, some studies concluded that BMI, WC, and WHtR could all be considered comparable in their association with hypertension [35–37]. A few other studies have suggested that BMI best predicted hypertension [16, 17]. In our studied population, we found that central obesity indicators performed better in predicting hypertension based on the results of correlation coefficients, AUC, and HR estimates. This may be because ectopic fat deposition triggers pathological metabolic responses that subsequently increase the risk of metabolic disease [40]. Excessive visceral fat distribution is accompanied by several alterations in hormonal, inflammatory and endothelial levels, and these alterations induce stimulation of several other mechanisms through increased insulin resistance, RAAS and sympathetic nervous system stimulation, impaired stress and chemoreflex cardiovascular control, endothelial dysfunction and increased sodium retention, leading to an increase in blood pressure levels [9]. Visceral fat distribution is influenced in part by genetic factors, which can also lead to elevated blood pressure levels in obese individuals (e.g., tumor necrosis factor-α, β3-adrenergic receptor, G-protein β3 subunit) [41].

Another potential explanation for the stronger association between central obesity indicators and hypertension could be the differences in the definitions of obesity by central obesity indicators versus general obesity indicators. Although BMI is the most widely used indicator of obesity, many studies have shown that BMI does not consider body fat distribution and there is some doubt concerning its ability to predict the accurate risk of hypertension [18, 42–44]. In contrast with BMI, WC, and WHtR are used as surrogate markers for body fat centralization [24, 39, 45]. Moreover, studies have shown that weight change is an indicator to assess changes in total fat mass, while WC change provides the measure of changes in body fat distribution. Most of the change in WC was attributable to changes in subcutaneous abdominal fat compared to weight change [46]. Central distribution of body fat is strongly associated with hypertension [39, 47, 48], and thus central obesity indicators performed better in predicting hypertension.

In addition, further comparisons of baseline to change measures in central obesity indicators based on correlation coefficients and AUC estimates were conducted to identify which indicators were more associated with hypertension. In general, although potential bias remains, we found that baseline central adiposity indicators (WC and WHtR) presented closer associations with hypertension than long-term WC change from baseline to follow-up. Yet after approximately 6.7 years of follow-up, the WC change had better predictive power for hypertension than baseline WHtR. This may be due to the accumulation of effects over time. And the study suggested that in contrast to baseline indices, long-term adiposity change may better capture the effects of excess fat because it affects individual differences in frame size and lean mass that are difficult to measure in population studies [49].

Strengths of this study include the availability of the first 10-year well-characterized population-based cohort in southwest China, whose relatively low loss to follow-up limited the potential bias of risk estimates. To our knowledge, this study is the first report to compare the predictive power of long-term weight change or waist circumference change on hypertension in southwest China. Our results provided a scientific basis for the prevention and control of hypertension development by changing poor lifestyles and suggested that more attention should be paid to waist circumference and central obesity. The present study also has some notable limitations. Firstly, only baseline information for most covariates was used in the regression analysis, which may cause residual confounding if those covariates may vary over time. Secondly, although the current regression analysis adjusted for the major potential confounders, residual confounding due to other confounding factors, such as dietary factors, cannot be excluded. Thirdly, the time of onset of hypertension in this cohort may be inaccurate because it was followed up only once. Fourthly, we could not account for the relationship between some typical obesity indicators (hip circumference, waist-to-hip ratio, and others) and the risk of hypertension due to the lack of data. Fifthly, the selection bias in lost to follow-up can lead to potential bias in our analysis and weaken the association between the obesity indicators and hypertension. Finally, the fact that our study was conducted only in Guizhou province limits the generalizability of our results to southwest China. And more studies should be done before any conclusions can be drawn.
