# Supplementary material for: Central Adiposity Indicators Maintain a Stronger Association With the Risk of Hypertension: A Prospective Cohort Study in Southwest China
Source: Int J Public Health. 2022 Oct 5;67:1605305. doi: 10.3389/ijph.2022.1605305 (PMC9579282; doi:10.3389/ijph.2022.1605305)
Supplement: Supplementary file 1 [file DataSheet1.docx]

**Supplementary Material: Central** **adiposity indicators maintain a stronger association with the risk of hypertension: a prospective cohort study in southwest China (International Journal of Public Health)**

**Supplementary Table S1.** Non-linear test results for the association between anthropometric measures and hypertension (The Guizhou population health cohort study, China, 2010-2012).

**Supplementary Table S2.** General characteristics of the study population at baseline in Southwest China (The Guizhou population health cohort study, China, 2010-2012).

**Supplementary Table S3.** Baseline characteristics between the complete follow-up and the lost to follow-up group in Southwest China (The Guizhou population health cohort study, China, 2010-2012).

**Supplementary Table S4.** Pearson correlation coefficients and 95% confidence intervals between anthropometric indices and blood pressure (The Guizhou population health cohort study, China, 2010-2012).

**Supplementary Table S5.** Areas under the receiver operating characteristic curves for the various anthropometric indices and hypertension (The Guizhou population health cohort study, China, 2010-2012).

**Supplementary Figure S1.** Time-dependent receiver operating characteristic curves for half of the follow-up person-years of waist-to-height ratio and waist circumference change from baseline to follow-up for hypertension (The Guizhou population health cohort study, China, 2010-2012).

**Supplementary Figure S2.** Time-dependent areas under curves of waist-to-height ratio and waist circumference change from baseline to follow-up for hypertension (The Guizhou population health cohort study, China, 2010-2012).

**Supplementary Table S6.** C-index of various Cox regression models (The Guizhou population health cohort study, China, 2010-2012).

**Supplementary Table S7.** Hazard ratios (95% confidence intervals) of hypertension associated with anthropometric indices after excluding new cases of hypertension within one year of follow-up (The Guizhou population health cohort study, China, 2010-2012).

**Supplementary Table S8.** E-values for the effect of anthropometric indices on hypertension (and its lower limit of 95% confidence intervals) in each adjusted Cox model (The Guizhou population health cohort study, China, 2010-2012).

**Supplementary Table S9.** Hazard ratios (95% confidence intervals) of hypertension associated with the measured covariates based on baseline body mass index, waist circumference, and waist-to-height ratio (The Guizhou population health cohort study, China, 2010-2012).

**Supplementary Table S10.** Hazard ratios (95% confidence intervals) of hypertension associated with the measured covariates based on weight change and waist circumference change from baseline to follow-up (The Guizhou population health cohort study, China, 2010-2012).

**Supplementary Table S1.** Non-linear test results for the association between anthropometric measures and hypertension (The Guizhou population health cohort study, China, 2010-2012).

|  | **BMI ^a^** | **WC ^a^** | **WHtR ^a^** | | **Weight Change** | **WC ^a^ Change** |
| --- | --- | --- | --- | --- | --- | --- |
| Model1 ^b^ | 0.59 | 0.71 | | 0.94 | 0.09 | 0.26 |
| Model2 ^c^ | 0.62 | 0.85 | | 0.98 | 0.08 | 0.43 |
| Model3 ^d^ | 0.67 | 0.86 | | 0.96 | 0.30 | 0.86 |

^a^ BMI: body mass index, WC: waist circumference, WHtR: waist-to-height ratio.

^b^ Adjusted for age (continuous variable), sex.

^c^ Model 1 plus area, ethnicity, marriage, occupation, smoking status, alcohol use, exercise, and history of diabetes.

^d^ Model 2 plus SBP, total cholesterol, triglycerides, HDL-C value, LDL-C value, and baseline BMI value (in the analyses of weight change and WC change).

**Supplementary Table S2.** General characteristics of the study population at baseline in Southwest China (The Guizhou population health cohort study, China, 2010-2012).

|  | **Total** | **No Hypertension** | **Hypertension** | ***P*-value** |
| --- | --- | --- | --- | --- |
| Participants, n | 5613 | 4399 | 1214 |  |
| **Demographic characteristics** |  |  |  |  |
| Rural, % | 3754 (66.9) | 2912 (66.2) | 842 (69.4) | 0.04 |
| Age at baseline, years | 42.02 ±14.17 | 40.59 ± 13.84 | 47.22 ± 14.14 | <0.001 |
| Men, % | 2559 (45.6) | 1969 (44.8) | 590 (48.6) | 0.02 |
| Ethnic minority, % | 2384 (42.5) | 1900 (43.2) | 484 (39.9) | 0.04 |
| Marriage, % |  |  |  | <0.001 |
| Married | 4522 (80.6) | 3518 (80.0) | 1004 (82.7) |  |
| Unmarried | 608 (10.8) | 530 (12.0) | 78 (6.4) |  |
| Others | 483 (8.6) | 351 (8.0) | 132 (10.9) |  |
| Occupation, % |  |  |  | <0.001 |
| Farmer | 3198 (57.0) | 2443 (55.5) | 755 (62.2) |  |
| Others | 1590 (28.3) | 1303 (29.6) | 287 (23.6) |  |
| Unemployed or retired | 825 (14.7) | 653 (14.8) | 172 (14.2) |  |
| Smoking status, % | 1516 (27.0) | 1151 (26.2) | 365 (30.1) | 0.01 |
| Alcohol use, % | 1735 (30.9) | 1353 (30.8) | 382 (31.5) | 0.66 |
| Exercise, % | 4851 (86.4) | 3797 (86.3) | 1054 (86.8) | 0.68 |
| History of diabetes, %^*^ | 354 (6.3) | 268 (6.1) | 86 (7.1) | 0.05 |
| **Biochemical characteristics** |  |  |  |  |
| Body mass index, kg/m^2^ | 22.51 ± 3.16 | 22.42 ± 3.15 | 22.86 ± 3.17 | <0.001 |
| <22.0 | 2722 (48.5) | 2189 (49.8) | 533 (43.9) | <0.001 |
| 22.0-23.9 | 1343 (23.9) | 1045 (23.8) | 298 (24.5) |  |
| 24.0-27.9 | 1277 (22.8) | 974 (22.1) | 303 (25.0) |  |
| ≥28.0 | 271 (4.8) | 191 (4.3) | 80 (6.6) |  |
| Waist circumference, cm^*^ | 75.32 ± 8.87 | 74.94 ± 8.71 | 76.67 ± 9.27 | <0.001 |
| ≥85/90 | 573 (10.9) | 405 (9.9) | 168 (14.5) | <0.001 |
| Waist-height ratio^*^ | 0.48 ± 0.06 | 0.48 ± 0.06 | 0.49 ± 0.06 | <0.001 |
| ≥0.5 | 1745 (33.1) | 1285 (31.3) | 460 (39.8) | <0.001 |
| Systolic blood pressure, mmHg^*^ | 116.20 ± 11.94 | 115.36 ± 11.87 | 119.25 ± 11.70 | <0.001 |
| Total cholesterol, mg/dL^*^ | 4.73 ± 1.30 | 4.71 ± 1.31 | 4.77 ± 1.25 | 0.17 |
| Triglycerides, mg/dL^*^ | 1.65 ± 1.50 | 1.61 ± 1.45 | 1.79 ± 1.65 | <0.001 |
| HDL cholesterol, mg/dL^*a^ | 1.45 ± 0.55 | 1.45 ± 0.52 | 1.46 ± 0.63 | 0.75 |
| LDL cholesterol, mg/dL^*a^ | 2.62 ± 1.17 | 2.63 ± 1.17 | 2.59 ± 1.15 | 0.38 |

^*^ Missing value.

^a^ HDL cholesterol: high-density lipoprotein cholesterol, LDL cholesterol: low-density lipoprotein cholesterol.

**Supplementary Table S3.** Baseline characteristics between the complete follow-up and the lost to follow-up group in Southwest China (The Guizhou population health cohort study, China, 2010-2012).

|  | **Total** | **Follow-up** | **Loss to follow-up** | | ***P*-value** |
| --- | --- | --- | --- | --- | --- |
| Participants, n | 9280 | 8163 | | 1117 |  |
| **Demographic characteristics** |  |  | |  |  |
| Rural, % | 6130 (66.1) | 5445 (66.7) | | 685 (61.3) | <0.001 |
| Age at baseline, years | 44.78±15.74 | 44.51±15.15 | | 46.78±19.42 | <0.001 |
| Men, % | 4442 (47.9) | 3872 (47.4) | | 570 (51.0) | 0.026 |
| Ethnic minority, % | 3750 (40.4) | 3372 (41.3) | | 378 (33.8) | <0.001 |
| Marriage, % |  |  | |  | <0.001 |
| Married | 7334 (79.0) | 6559 (80.4) | | 775 (69.4) |  |
| Unmarried | 959 (10.3) | 773 (9.5) | | 186 (16.7) |  |
| Others | 987 (10.6) | 831 (10.2) | | 156 (14.0) |  |
| Occupation, % |  |  | |  | <0.001 |
| Farmer | 5226 (56.3) | 4654 (57.0) | | 572 (51.2) |  |
| Others | 2472 (26.6) | 2185 (26.8) | | 287 (25.7) |  |
| Unemployed or retired | 1582 (17.0) | 1324 (16.2) | | 258 (23.1) |  |
| Smoking status, % | 2690 (29.0) | 2336 (28.6) | | 354 (31.7) | 0.037 |
| Alcohol use, % | 2992 (32.2) | 2625 (32.2) | | 367 (32.9) | 0.664 |
| Exercise, % | 8033 (86.6) | 7097 (86.9) | | 936 (83.8) | 0.004 |
| History of diabetes, %^*^ | 809 (8.7) | 689 (8.4) | | 120 (10.7) | 0.033 |
| **Biochemical characteristics** |  |  | |  |  |
| Body mass index, kg/m^2^ | 22.83 ± 3.34 | 22.88 ± 3.35 | | 22.44 ± 3.23 | <0.001 |
| <22.0 | 4181 (45.2) | 3617 (44.4) | | 564 (50.7) | <0.001 |
| 22.0-23.9 | 2158 (23.3) | 1917 (23.5) | | 241 (21.7) |  |
| 24.0-27.9 | 2277 (24.6) | 2037 (25.0) | | 240 (21.6) |  |
| ≥28.0 | 644 (7.0) | 577 (7.1) | | 67 (6.0) |  |
| Waist circumference, cm^*^ | 76.51 ± 9.43 | 76.56 ± 9.46 | | 76.15 ± 9.21 | 0.181 |
| ≥85/90 | 1249 (14.4) | 1100 (14.5) | | 149 (14.2) | 0.854 |
| Waist-height ratio^*^ | 0.49 ± 0.06 | 0.49 ± 0.06 | | 0.48 ± 0.06 | 0.069 |
| ≥0.5 | 3338 (38.6) | 2942 (38.7) | | 396 (37.8) | 0.603 |
| Systolic blood pressure, mmHg^*^ | 125.84 ± 21.57 | 125.49 ± 21.14 | | 128.40 ± 24.38 | <0.001 |
| Total cholesterol, mg/dL^*^ | 4.76 ± 1.33 | 4.79 ± 1.32 | | 4.61 ± 1.39 | <0.001 |
| Triglycerides, mg/dL^*^ | 1.75 ± 1.55 | 1.75 ± 1.56 | | 1.75 ± 1.51 | 0.939 |
| HDL cholesterol, mg/dL^*a^ | 1.45 ± 0.65 | 1.45 ± 0.57 | | 1.45 ± 1.04 | 0.878 |
| LDL cholesterol, mg/dL^*a^ | 2.61 ± 1.19 | 2.65 ± 1.18 | | 2.35 ± 1.21 | <0.001 |

^*^ Missing value.

^a^ HDL cholesterol: high-density lipoprotein cholesterol, LDL cholesterol: low-density lipoprotein cholesterol.

**Supplementary Table S4.** Pearson correlation coefficients and 95% confidence intervals between anthropometric indices and blood pressure (The Guizhou population health cohort study, China, 2010-2012).

|  | **BMI ^a^** | **WC ^a^** | **WHtR ^a^** | | **Weight Change** | **WC ^a^ Change** |
| --- | --- | --- | --- | --- | --- | --- |
| SBP ^a^ | 0.17 (0.15, 0.20) * | **0.18** **(0.15, 0.20) ^b^** | | 0.14 (0.11, 0.16) | **-0.08 (-0.11, -0.05)** | -0.06 (-0.09, -0.03) |
| DBP ^a^ | 0.21 (0.18, 0.23) | **0.24 (0.22, 0.27)** | | 0.19 (0.17, 0.22) | -0.08 (-0.11, -0.05) | **-0.11 (-0.14, -0.08)** |

^a^ SBP: systolic blood pressure, DBP: diastolic blood pressure, BMI: body mass index, WC: waist circumference, WHtR: waist-to-height ratio.

^b^ The anthropometric measure with the highest correlation coefficient for each blood pressure was marked in **bold** (separate comparisons of baseline and change measures).

* All are significant at the level of <0.001 (2-tailed).

**Supplementary Table S5.** Areas under the receiver operating characteristic curves for the various anthropometric indices and hypertension (The Guizhou population health cohort study, China, 2010-2012).

|  | **BMI ^a^** | **WC ^a^** | **WHtR ^a^** | **Weight Change** | **WC ^a^ Change** |
| --- | --- | --- | --- | --- | --- |
| AUC (95%CI) ^a^ | 0.57 (0.55, 0.59) | 0.58 (0.56, 0.61) | **0.59 (0.57, 0.61) ^b^** | 0.54 (0.51, 0.56) | **0.58 (0.56, 0.61)** |

^a^ BMI: body mass index, WC: waist circumference, WHtR: waist-to-height ratio, AUC: areas under the receiver operating characteristic curves, 95%CI: 95% confidence interval.

^b^ The anthropometric measure with the highest AUC value was marked in **bold** (separate comparisons of baseline and change measures)**.**


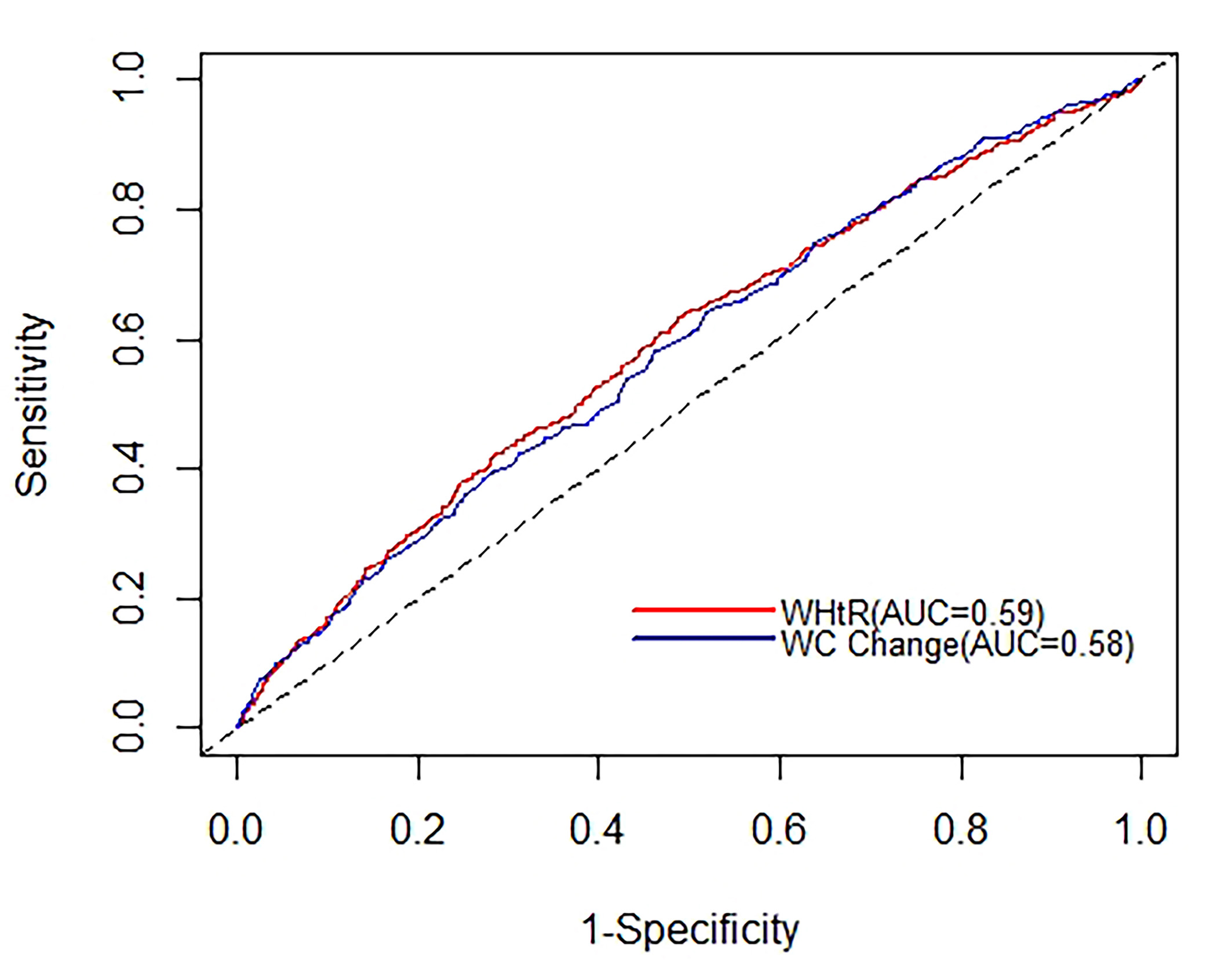


**Supplementary Figure S1.** Time-dependent receiver operating characteristic curves for half of the follow-up person-years of waist-to-height ratio and waist circumference change from baseline to follow-up for hypertension (The Guizhou population health cohort study, China, 2010-2012).

WHtR: waist-to-height ratio, WC: waist circumference.


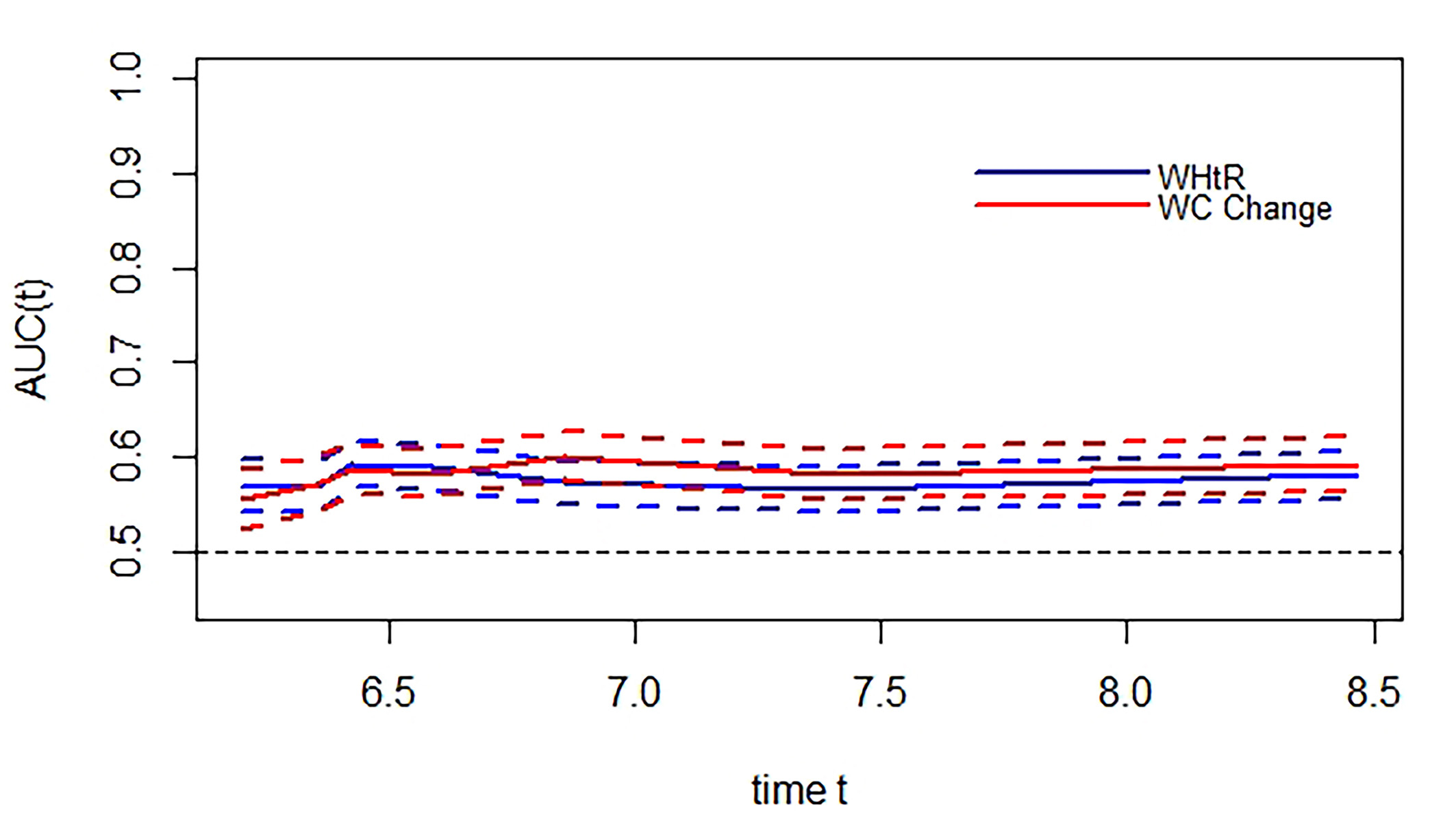


**Supplementary Figure S2.** Time-dependent areas under curves of waist-to-height ratio and waist circumference change from baseline to follow-up for hypertension (The Guizhou population health cohort study, China, 2010-2012).

WHtR: waist-to-height ratio, WC: waist circumference, AUC: areas under the receiver operating characteristic curves.

**Supplementary Table S6.** C-index of various Cox regression models (The Guizhou population health cohort study, China, 2010-2012).

|  | **C-index ^a^** | | |
| --- | --- | --- | --- |
|  | **Model 1 ^b^** | **Model 2 ^c^** | **Model 3 ^d^** |
| Body mass index, kg/m^2^ | 0.651 | 0.658 | 0.671 |
| Waist circumference, cm | **0.651** | **0.660** | **0.671** |
| Waist-to-height ratio | 0.650 | 0.660 | 0.671 |
| Weight change, kg | 0.655 | 0.665 | 0.690 |
| Waist circumference change, cm | **0.665** | **0.677** | **0.698** |

^a^ C-index: Harrell' concordance index.

^b^ Adjusted for age (continuous variable), sex.

^c^ Model 1 plus area, ethnicity, marriage, occupation, smoking status, alcohol use, exercise, and history of diabetes.

^d^ Model 2 plus SBP, total cholesterol, triglycerides, HDL-C value, LDL-C value, and baseline BMI value (in the analyses of weight change and WC change).

*Anthropometric measures with the highest C-index value in three models were marked in bold (separate comparisons of baseline and change measures).

**Supplementary Table S7.** Hazard ratios (95% confidence intervals) of hypertension associated with anthropometric indices after excluding new cases of hypertension within one year of follow-up (The Guizhou population health cohort study, China, 2010-2012).

|  | **Cases, n** | **HR (95%CI) ^a^** | | |
| --- | --- | --- | --- | --- |
|  |  | **Model 1 ^b^** | **Model 2 ^c^** | **Model 3 ^d^** |
| **Body mass index, kg/m^2^** | 5578 | 1.15 (1.09, 1.21) ^***^ | 1.14 (1.07, 1.20) ^***^ | 1.10 (1.03, 1.17) ^**^ |
| <22.0 | 2709 | 0.85 (0.74, 0.98) ^*^ | 0.86 (0.74, 0.99) ^*^ | 0.91 (0.79, 1.06) |
| 22.0-23.9 | 1331 | 1.00 | 1.00 | 1.00 |
| 24.0-27.9 | 1268 | 1.01 (0.86, 1.19) | 1.00 (0.85, 1.18) | 0.99 (0.84, 1.17) |
| ≥28.0 | 270 | 1.62 (1.26, 2.07) ^***^ | 1.56 (1.21, 2.00) ^***^ | 1.52 (1.17, 1.96) ^**^ |
| **Waist circumference, cm** | 5234 | 1.19 (1.12, 1.26) ^***^ | 1.18 (1.11, 1.26) ^***^ | 1.15 (1.08, 1.22) ^***^ |
| <85/90 | 4666 | 1.00 | 1.00 | 1.00 |
| ≥85/90 | 568 | 1.42 (1.21, 1.68) ^***^ | 1.40 (1.18, 1.66) ^***^ | 1.31 (1.10, 1.55) ^**^ |
| **Waist-to-height ratio** | 5234 | 1.17 (1.11, 1.24) ^***^ | 1.17 (1.10, 1.24) ^***^ | 1.14 (1.07, 1.21) ^***^ |
| <0.5 | 3502 | 1.00 | 1.00 | 1.00 |
| ≥0.5 | 1732 | 1.31 (1.16, 1.48) ^***^ | 1.29 (1.14, 1.46) ^***^ | 1.24 (1.09, 1.40) ^**^ |
| **Weight change, kg** | 4455 | 1.18 (1.11, 1.26) **^***^** | 1.18 (1.11, 1.26) **^***^** | 1.26 (1.18, 1.34) **^***^** |
| loss of > 2 | 1059 | 0.96 (0.79, 1.17) | 0.96 (0.79, 1.17) | 0.90 (0.74, 1.09) |
| loss of ≤2 to gain of < 2 | 1094 | 1.00 | 1.00 | 1.00 |
| gain of ≥2 to gain of < 6 | 996 | 1.35 (1.12, 1.64) ^**^ | 1.32 (1.09, 1.60) ^**^ | 1.40 (1.15, 1.70) ^***^ |
| gain of ≥6 | 1306 | 1.37 (1.14, 1.63) ^***^ | 1.37 (1.14, 1.64) ^***^ | 1.52 (1.26, 1.83) ^***^ |
| **Waist circumference change, cm** | 4060 | 1.33 (1.25, 1.42) **^***^** | 1.32 (1.24, 1.40) **^***^** | 1.41 (1.32, 1.51) **^***^** |
| loss of > 3 | 605 | 0.85 (0.66, 1.09) | 0.84 (0.65, 1.08) | 0.77 (0.60, 1.00) ^*^ |
| loss of ≤3 to gain of < 3 | 900 | 1 | 1 |  |
| gain of ≥3 to gain of < 9 | 1020 | 1.27 (1.03, 1.57) ^*^ | 1.27 (1.03, 1.56) ^*^ | 1.38 (1.12, 1.71) ^**^ |
| gain of ≥ 9 | 1535 | 1.64 (1.37, 1.98) ^***^ | 1.59 (1.32, 1.92) ^***^ | 1.77 (1.46, 2.15) ^***^ |

^a^ HR: hazard ratio, 95%CI: 95% confidence interval.

^b^ Adjusted for age (continuous variable), sex.

^c^ Model 1 plus area, ethnicity, marriage, occupation, smoking status, alcohol use, exercise, and history of diabetes.

^d^ Model 2 plus SBP, total cholesterol, triglycerides, HDL-C value, LDL-C value, and baseline BMI value (in the analyses of weight change and WC change).

^***^ *P*<0.001, ^**^ *P*<0.01, ^*^ *P*<0.05.

**Supplementary Table S8.** E-values for the effect of anthropometric indices on hypertension (and its lower limit of 95% confidence intervals) in each adjusted Cox model (The Guizhou population health cohort study, China, 2010-2012).

| **Model** | **E-value for HR ^d^ estimate** | **E-value for**  **lower limit of**  **95% CI ^d^** | **Variable** | **Level** | **HR (95% CI) ^d^** |
| --- | --- | --- | --- | --- | --- |
| Model1 ^a^ | 2.09 | 1.59 | BMI ^d^, kg/m2 | ≥28.0 vs. 22.0-23.9 | 1.58 (1.24, 2.03) |
|  | 1.88 | 1.54 | WC ^d^, cm | ≥85/90 vs. <85/90 | 1.43 (1.21, 1.69) |
|  | 1.70 | 1.47 | WHtR ^d^ | ≥0.5 vs. <0.5 | 1.31 (1.17, 1.48) |
|  | 1.78  1.84 | 1.38  1.47 | Weight change, kg | gain of ≥2 to gain of < 6 vs. loss of ≤2 to gain of < 2  gain of ≥6 vs. loss of ≤2 to gain of < 2 | 1.36 (1.12, 1.64)  1.40 (1.17, 1.67) |
|  | 1.63 | 1.17 | WC ^d^ change, cm | gain of ≥3 to gain of < 9 vs. loss of ≤3 to gain of < 3 | 1.26 (1.03, 1.55) |
|  | 2.23 | 1.85 |  | gain of ≥ 9 vs. loss of ≤3 to gain of < 3 | 1.69 (1.41, 2.03) |
| Model2 ^b^ | 2.02 | 1.51 | BMI, kg/m2 | ≥28.0 vs. 22.0-23.9 | 1.53 (1.19, 1.96) |
|  | 1.85 | 1.53 | WC, cm | ≥85/90 vs. <85/90 | 1.41 (1.20, 1.67) |
|  | 1.69 | 1.44 | WHtR | ≥0.5 vs. <0.5 | 1.30 (1.15, 1.46) |
|  | 1.73  1.85 | 1.34  1.49 | Weight change, kg | gain of ≥2 to gain of < 6 vs. loss of ≤2 to gain of < 2  gain of ≥6 vs. loss of ≤2 to gain of < 2 | 1.33 (1.10, 1.61)  1.41 (1.18, 1.68) |
|  | 1.63  2.17 | 1.13  1.78 | WC change, cm | gain of ≥3 to gain of < 9 vs. loss of ≤3 to gain of < 3  gain of ≥ 9 vs. loss of ≤3 to gain of < 3 | 1.26 (1.02, 1.55)  1.64 (1.36, 1.97) |
| Model3 ^c^ | 1.98 | 1.45 | BMI, kg/m2 | ≥28.0 vs. 22.0-23.9 | 1.50 (1.16, 1.93) |
|  | 1.73 | 1.38 | WC, cm | ≥85/90 vs. <85/90 | 1.33 (1.12, 1.57) |
|  | 1.61 | 1.34 | WHtR | ≥0.5 vs. <0.5 | 1.25 (1.10, 1.42) |
|  | 1.84  2.06 | 1.45  1.67 | Weight change, kg | gain of ≥2 to gain of < 6 vs. loss of ≤2 to gain of < 2  gain of ≥6 vs. loss of ≤2 to gain of < 2 | 1.40 (1.16, 1.70)  1.56 (1.29, 1.87) |
|  | 1.79  2.40 | 1.36  1.99 | WC change, cm | gain of ≥3 to gain of < 9 vs. loss of ≤3 to gain of < 3  gain of ≥ 9 vs. loss of ≤3 to gain of < 3 | 1.37 (1.11, 1.69)  1.83 (1.51, 2.21) |

^a^ Adjusted for age (continuous variable), sex.

^b^ Model 1 plus area, ethnicity, marriage, occupation, smoking status, alcohol use, exercise, and history of diabetes.

^c^ Model 2 plus SBP, total cholesterol, triglycerides, HDL-C value, LDL-C value, and baseline BMI value (in the analyses of weight change and WC change).

^d^ BMI: body mass index, WC: waist circumference, WHtR: waist-to-height ratio, HR: hazard ratio, 95%CI: 95% confidence interval.

**Supplementary Table S9.** Hazard ratios (95% confidence intervals) of hypertension associated with the measured covariates based on baseline body mass index, waist circumference, and waist-to-height ratio (The Guizhou population health cohort study, China, 2010-2012).

| **Covariates** | **Level** | **HR (95%CI) ^a^** | | |
| --- | --- | --- | --- | --- |
|  |  | **Model 1 ^b^** | **Model 2 ^c^** | **Model 3 ^d^** |
| Age | Age | 1.03 (1.03, 1.04) | 1.03 (1.03, 1.04) | 1.03 (1.03, 1.04) |
| Sex | Men vs. Women | 1.23 (1.10, 1.38) | 1.24 (1.06, 1.45) | 1.19 (1.02, 1.40) |
| Area | Rural vs. Urban | 0.75 (0.66, 0.86) | 0.67 (0.58, 0.77) | 0.70 (0.60, 0.82) |
| Ethnicity | Yes vs. No | 1.03 (0.91, 1.16) | 1.14 (1.00, 1.29) | 1.20 (1.05, 1.38) |
| Marriage | Unmarried vs. Married | 0.85 (0.66, 1.10) | 0.88 (0.68, 1.13) | 0.86 (0.67, 1.12) |
|  | Others vs. Married | 1.03 (0.85, 1.24) | 1.03 (0.86, 1.25) | 1.02 (0.84, 1.24) |
| Occupation | Others vs. Farmer | 0.87 (0.75, 1.00) | 0.79 (0.68, 0.92) | 0.81 (0.70, 0.95) |
|  | Unemployed or retired vs. Farmer | 0.86 (0.73, 1.02) | 0.82 (0.68, 0.98) | 0.81 (0.68, 0.97) |
| Smoking status | Yes vs. No | 1.05 (0.89, 1.24) | 0.96 (0.81, 1.14) | 0.96 (0.81, 1.14) |
| Alcohol use | Yes vs. No | 1.12 (0.98, 1.29) | 1.09 (0.94, 1.26) | 1.08 (0.93, 1.25) |
| Exercise | Yes vs. No | 1.36 (1.14, 1.62) | 1.23 (1.02, 1.47) | 1.14 (0.95, 1.37) |
| History of diabetes | Yes vs. No | 0.93 (0.73, 1.17) | 0.94 (0.75, 1.19) | 0.92 (0.73, 1.16) |
| SBP ^a^ | SBP | 1.01 (1.00, 1.01) | 1.01 (1.00, 1.01) | 1.01 (1.00, 1.02) |
| Triglycerides | Triglycerides | 1.00 (0.97, 1.04) | 1.00 (0.96, 1.04) | 1.04 (1.00, 1.08) |
| Total cholesterol | Total cholesterol | 0.96 (0.92, 1.01) | 0.96 (0.91, 1.00) | 0.83 (0.77, 0.90) |
| HDL cholesterol ^a^ | HDL cholesterol | 1.01 (0.92, 1.11) | 0.96 (0.87, 1.06) | 1.09 (0.98, 1.22) |
| LDL cholesterol ^a^ | LDL cholesterol | 1.06 (1.01, 1.11) | 1.05 (0.99, 1.10) | 1.20 (1.11, 1.29) |

^a^ HR: hazard ratio, 95%CI: 95% confidence interval, SBP: systolic blood pressure, HDL cholesterol: high-density lipoprotein cholesterol, LDL cholesterol: low-density lipoprotein cholesterol.

^b^ Adjusted for age (continuous variable), sex, and HRs (95%CIs) for age and sex are in the original model.

^c^ Model 1 plus area, ethnicity, marriage, occupation, smoking status, alcohol use, exercise, and history of diabetes, and HRs (95%CIs) for age, sex, area, ethnicity, marriage, occupation, smoking status, alcohol use, exercise, and history of diabetes are in the original model.

^d^ Model 2 plus SBP, total cholesterol, triglycerides, HDL-C value, LDL-C value.

**Supplementary Table S10.** Hazard ratios (95% confidence intervals) of hypertension associated with the measured covariates based on weight change and waist circumference change from baseline to follow-up (The Guizhou population health cohort study, China, 2010-2012).

| **Covariates** | **Level** | **HR (95%CI) ^a^** | | |
| --- | --- | --- | --- | --- |
|  |  | **Model 1 ^b^** | **Model 2 ^c^** | **Model 3 ^d^** |
| Age | Age | 1.04 (1.03, 1.04) | 1.03 (1.03, 1.04) | 1.03 (1.03, 1.04) |
| Sex | Men vs. Women | 1.16 (1.02, 1.32) | 1.15 (0.97, 1.37) | 1.10 (0.92, 1.32) |
| Area | Rural vs. Urban | 0.75 (0.65, 0.86) | 0.67 (0.57, 0.79) | 0.71 (0.60, 0.85) |
| Ethnicity | Yes vs. No | 0.94 (0.82, 1.08) | 1.05 (0.90, 1.22) | 1.12 (0.96, 1.30) |
| Marriage | Unmarried vs. Married | 0.93 (0.71, 1.23) | 0.93 (0.71, 1.23) | 0.97 (0.73, 1.28) |
|  | Others vs. Married | 1.05 (0.84, 1.30) | 1.05 (0.84, 1.31) | 1.02 (0.81, 1.27) |
| Occupation | Others vs. Farmer | 0.86 (0.73, 1.00) | 0.76 (0.64, 0.90) | 0.78 (0.65, 0.93) |
|  | Unemployed or retired vs. Farmer | 0.92 (0.76, 1.10) | 0.82 (0.67, 1.00) | 0.83 (0.68, 1.01) |
| Smoking status | Yes vs. No | 1.07 (0.89, 1.29) | 0.99 (0.82, 1.20) | 1.01 (0.84, 1.23) |
| Alcohol use | Yes vs. No | 1.08 (0.92, 1.25) | 1.05 (0.90, 1.23) | 1.03 (0.88, 1.21) |
| Exercise | Yes vs. No | 1.28 (1.06, 1.54) | 1.15 (0.94, 1.39) | 1.05 (0.86, 1.28) |
| History of diabetes | Yes vs. No | 0.99 (0.78, 1.27) | 1.00 (0.78, 1.27) | 0.88 (0.69, 1.13) |
| SBP ^a^ | BMI | 1.07 (1.05, 1.09) | 1.07 (1.05, 1.09) | 1.06 (1.04, 1.08) |
| Triglycerides | SBP | 1.01 (1.01, 1.02) | 1.01 (1.01, 1.02) | 1.01 (1.01, 1.02) |
| Total cholesterol | Triglycerides | 1.04 (1.01, 1.08) | 1.04 (1.01, 1.08) | 1.05 (1.01, 1.09) |
| HDL cholesterol ^a^ | Total cholesterol | 0.99 (0.94, 1.04) | 0.99 (0.94, 1.04) | 0.89 (0.81, 0.97) |
| LDL cholesterol ^a^ | HDL cholesterol | 0.94 (0.84, 1.05) | 0.90 (0.80, 1.01) | 1.01 (0.89, 1.16) |
| Age | LDL cholesterol | 1.07 (1.02, 1.13) | 1.05 (1.00, 1.11) | 1.14 (1.05, 1.23) |

^a^ HR: hazard ratio, 95%CI: 95% confidence interval, SBP: systolic blood pressure, HDL cholesterol: high-density lipoprotein cholesterol, LDL cholesterol: low-density lipoprotein cholesterol.

^b^ Adjusted for age (continuous variable), sex, and HRs (95%CIs) for age and sex are in the original model.

^c^ Model 1 plus area, ethnicity, marriage, occupation, smoking status, alcohol use, exercise, and history of diabetes, and HRs (95%CIs) for age, sex, area, ethnicity, marriage, occupation, smoking status, alcohol use, exercise, and history of diabetes are in the original model.

^d^ Model 2 plus SBP, total cholesterol, triglycerides, HDL-C value, LDL-C value, baseline BMI value.
